# Interference between Redroot Pigweed (*Amaranthus retroflexus* L.) and Cotton (*Gossypium hirsutum* L.): Growth Analysis

**DOI:** 10.1371/journal.pone.0130475

**Published:** 2015-06-09

**Authors:** Xiaoyan Ma, Hanwen Wu, Weili Jiang, Yajie Ma, Yan Ma

**Affiliations:** 1 State Key Laboratory of Cotton Biology, Institute of Cotton Research, Chinese Academy of Agricultural Sciences, Anyang, China; 2 Graham Centre for Agricultural Innovation, Wagga Wagga Agricultural Institute, NSW Department of Primary Industries, Wagga Wagga, NSW, Australia; East Carolina University, UNITED STATES

## Abstract

Redroot pigweed is one of the injurious agricultural weeds on a worldwide basis. Understanding of its interference impact in crop field will provide useful information for weed control programs. The effects of redroot pigweed on cotton at densities of 0, 0.125, 0.25, 0.5, 1, 2, 4, and 8 plants m^-1^ of row were evaluated in field experiments conducted in 2013 and 2014 at Institute of Cotton Research, CAAS in China. Redroot pigweed remained taller and thicker than cotton and heavily shaded cotton throughout the growing season. Both cotton height and stem diameter reduced with increasing redroot pigweed density. Moreover, the interference of redroot pigweed resulted in a delay in cotton maturity especially at the densities of 1 to 8 weed plants m^-1^ of row, and cotton boll weight and seed numbers per boll were reduced. The relationship between redroot pigweed density and seed cotton yield was described by the hyperbolic decay regression model, which estimated that a density of 0.20–0.33 weed plant m^-1^ of row would result in a 50% seed cotton yield loss from the maximum yield. Redroot pigweed seed production per plant or per square meter was indicated by logarithmic response. At a density of 1 plant m^-1^ of cotton row, redroot pigweed produced about 626,000 seeds m^-2^. Intraspecific competition resulted in density-dependent effects on weed biomass per plant, a range of 430–2,250 g dry weight by harvest. Redroot pigweed biomass ha^-1^ tended to increase with increasing weed density as indicated by a logarithmic response. Fiber quality was not significantly influenced by weed density when analyzed over two years; however, the fiber length uniformity and micronaire were adversely affected at density of 1 weed plant m^-1^ of row in 2014. The adverse impact of redroot pigweed on cotton growth and development identified in this study has indicated the need of effective redroot pigweed management.

## Introduction

Redroot pigweed (*Amaranthus retroflexus* L.), an annual C4 weed, is listed as one of the most common dicotyledonous weeds in the world and is widely distributed in many agricultural areas [1]. The presence of redroot pigweed can severely reduce the yield of several crops, such as beans (*Phaseolus* spp.) [2–4], soybean (*Glycine max*) [5], corn (*Zea mays*) [6–8], and cotton (*Gossypium hirsutum*) [9, 10]. Redroot pigweed has been considered among the top-ranking troublesome weeds in many parts of the world [11] due, in part, to its prolific seed production [12], prolonged seed longevity [13, 14] and extended germination period [15], which allow redroot pigweed to form a persistent soil seed bank. Moreover, the vigorous aggressive growth and allelopathic activity of redroot pigweed plants allow them to compete strongly against other plants and significantly reduce crop yield and quality [16–18]. To date, redroot pigweed has been confirmed to have evolved resistance to either acetolactate synthase (ALS) inhibitors or Photosystem II inhibitors [19], which make it challenging to control in cropping system.

Considerable research has been reported on the interference and competitiveness of *Amaranthus* spp. species in cotton field, including tumble pigweed (*A*. *albus*), Palmer amaranth (*A*. *palmeri*), and redroot pigweed. Rushing *et al*. [20] reported that the threshold density where initial cotton yield reductions occurred at 4–16 tumble pigweed plants 10 m^-1^ of row and cotton yields were reduced 8–11 kg ha^-1^ for each additional weed plant 10 m^-1^ of crop row. Previous studies demonstrated maximum cotton yield losses due to full season interference of Palmer amaranth were 22% at 0.3 weed plant m^-1^ of row [21] and 54% at 1.1 plants m^-1^ of row [22]. MacRae *et al*. [23] found that Palmer amaranth competition could result in a significant reduction (60%) in cotton yield at an approximate density of 1.6 weed plants m^-1^ of row when weed plants were transplanted into 3- or 8-leaf cotton. The cotton yield loss due to redroot pigweed competition was reported to be 5–90% depending on the weed density and soil pattern [9]. Another study indicated that cotton yield and redroot pigweed density were highly correlated and the cotton yield loss ranged from 21 to 38 kg ha^-1^ for each redroot pigweed plant 15 m^-1^ of row [10].

Previous weed interference studies have indicated that cotton is sensitive to the competition of weeds and its vegetative growth is generally less sensitive than cotton yield and yield components when exposed to weed competition [24, 25]. Some previous studies showed that cotton vegetative growth was not affected by weed competition [26–30]. However, the adverse impacts on cotton growth, e.g. cotton plant height and stem diameter, due to weed competition have also been reported [9, 31–34]. In addition, the effect of weed species on cotton growth and development process has also been evaluated. Barnett and Steckel [33] indicated that some lint yield loss caused by giant ragweed (*Ambrosia trifida*) may have resulted from a delay in cotton maturity based on node above white flower and node above cracked boll data. Another study in China found that, under the competition of coastalplain yellowtops (*Flaveria bidentis*), the cotton budding stage was postponed due to increased weed densities. Meanwhile, sympodial branch number and square and boll number were also reduced [35]. So far, there is limited information on the effect of interference between redroot pigweed and cotton on plant growth and productivity. Therefore, the objectives of this study were to evaluate the effect of redroot pigweed densities on cotton growth and development parameters, yield, and fiber properties and to evaluate redroot pigweed growth and seed production at several densities in cotton.

## Materials and Methods

### Field arrangement

Field studies were conducted from April to October in 2013 and 2014 at the research farm of Institute of Cotton Research, CAAS (36.13° N, 114.85° E) in the Yellow River cotton-producing area of China. The soil was a sandy loam with a pH of 8.0 and an organic matter content of 1.5%. Local meteorological data during the experimental periods are presented in Table 1, and the site is characterized by wet and hot summers from May to August. Seeds of redroot pigweed were collected in autumn at the experimental fields and stored at room temperature until planting in the next spring.

**Table 1:** Monthly mean temperature and total precipitation from April to October for 2013 and 2014 at the experimental site (Anyang Meteorological Bureau).

Month	Temperature		Precipitation	
	2013	2014	2013	2014
	°C		mm	
April	14.1	16.0	5.7	45.6
May	21.6	22.8	64.3	31.8
June	25.8	26.0	76.5	37.2
July	27.3	26.9	226.0	129.4
August	28.0	25.1	36.6	46.1
September	21.5	20.6	16.0	156.1
October	15.4	16.7	10.6	4.3
Total	/	/	435.7	450.5

During both study years, tillage practices consisted of winter moldboard plowing followed by irrigating, disking and harrowing in spring. The experimental field was fertilized with 1,500 kg ha^-1^ compound fertilizer (N-P_2_O_5_-K_2_O = 24-11-5, ≥ 40%; Zhengzhou Naweigao Fertilizer Co., Ltd, Henan Province, China) prior to planting. CCRI 79, a commonly grown cotton cultivar in the region, was sown by hand at about 200 seeds 8 m^-1^ of row on 27 April 2013 and 1 May 2014. Plots were 8 m long and four 80-cm rows wide. Seeds of redroot pigweed were hand planted in hills at densities of 0, 1, 2, 4, 8, 16, 32, and 64 plants 8 m^-1^ of cotton row, or to 0, 0.125, 0.25, 0.5, 1, 2, 4 and 8 plants m^-1^ of row in the center two rows of each plot. Seeds were sowed approximately 1 cm deep and 10 cm away from the cotton row at desired intervals immediately after cotton planting. The outside rows of each plot were weed free and served as border rows between adjacent plots. The weeds emerged simultaneously with cotton plants (approximately 10 d after sowing). Cotton seedlings were thinned at the 3–4 leaf stage to about 4 seedlings m^-1^ of row (50,000 cotton plants ha^-1^) and redroot pigweed seedlings also were thinned at the 5–6 leaf stage to obtain the final required density. The experimental design was a randomized complete block with four replications. 150 kg ha^-1^ urea (N≥ 46.4%; Anyang Chemical Industry Co., Ltd, Henan Province, China) and 300 kg ha^-1^ compound fertilizer were used in mid-June and mid-July to optimize cotton growth, and insect and disease control practices were applied as required. No herbicides or irrigation treatments were used during these experiments. All weeds other than redroot pigweed were removed at approximately weekly intervals throughout the growing season by hand-hoeing.

### Plant survey

Cotton plant height, stem diameter, and square, bloom, and boll number per plant were measured from five randomly selected plants in the center 6 m of the center two rows at each plot. The measurements were carried out once a half month from the cotton seedling stage to the preharvest stage each year, totally for 8 times in 2013 [34, 49, 65, 79, 97, 110, 131, and 141 d after planting (DAP)] and in 2014 (42, 57, 71, 89, 102, 118, 132, and 148 DAP). Height was measured in cm from the soil surface to the apical meristem, and stem diameter was determined at the soil line with calipers to the nearest 0.01 mm. Bolls were graded, including small bolls (being wrapped in bract and < 2 cm in diameter), large bolls (developing out bract and > 2 cm in diameter), and cracked bolls, and recorded respectively. Two to five redroot pigweed plants were selected randomly for measurement of plant height and stem diameter each time. Upon maturity (3 September in 2013 and 22 September in 2014), redroot pigweed plants were removed from plots and 2–5 randomly selected redroot pigweed plants from each plot were cut at ground level with pruning shears, dried at 70°C for 48 h and weighed to determine the individual weed dry biomass. In 2014, seed productions of these 2–5 selected redroot pigweed plants were determined. All inflorescences per plant were clipped and oven-dried at 70°C for 48 h. Seeds were removed from plant debris and perianth by hand rubbing, and then seeds were cleaned with an air cleaner to remove debris and perianth. The cleaned seeds were weighed, and about four 0.1-g samples were taken and seed numbers counted. The total number of seeds per plant was then estimated by multiplying the ratio of bulk seeds weight to sample seed weight times the number of seed in each sample.

At the end of the growing season, cotton of the center two rows was hand-harvested twice from each plot, first at 50% open bolls and the second at 100% open bolls. Weights of the total hand-harvested cotton were recorded. Immediately before cotton harvest, one mature boll of middle branch was harvested from each of 10 randomly selected plants in each plot and was ginned together on a small single roller gin to determine boll weight, lint percentage, seed number per boll and seed index. Lint percentage is an expression of the ratio of the weight of the lint to the total weight of the seed cotton. The seed was acid delinted prior to weight determination. Four lots of 100-seed random samples were collected from each plot and measured, and the average weight was regarded as the seed index. After the measurement, a 25-g lint sample was subjected to fiber quality tests, which included fiber length, length uniformity, micronaire, breaking elongation and fiber strength, at the Supervision, Inspection and Test Center of Cotton Quality, Ministry of Agriculture, China.

### Statistical analyses

All data were analyzed using the general linear models (GLMs) treating redroot pigweed density and year as fixed factors to test for significant main effects and interactions. Since there were significant weed density by year interactions, data were separately analyzed by year. ANOVA and Fisher’s Protected LSD tests were used to determine variation among treatments. Regression analyses were performed to analyze the relationships between redroot pigweed density (plant m^-1^ of row) and (1) cotton plant height and stem diameter, (2) weed dry biomass, including individual plant biomass (g plant^-1^) and total dry biomass (kg ha^-1^), and (3) weed seed production, including seed production per plant (seeds plant^-1^) and total seed production (seeds m^-2^). These relationships were established from the best fits of the experimental data to appropriate functions, and coefficients of determination (*r* ^*2*^) were reported to indicate the amount of variation in the dependent variables that can be explained by the independent variable.

The Gompertz equation [Eq 1; 26] was fit to plant heights and stem diameters of each species in each plot over the growing season:

$$Y=a\;e^{be^{kT}}$$ (1)

where *Y* is the plant height in centimeters or stem diameter in millimeters, a is the upper asymptote for late-season plant height or stem diameter, *b* and *k* are constants, *e* is the base of the natural logarithms, and *T* is the time in days after planting.

A two-parameter hyperbolic decay regression model [Eq 2; 33] was used to describe the density-dependent effects of redroot pigweed on seed cotton yield:

Y=ab/(b+D) (2)

where *Y* is the seed cotton yield, *a* is the asymptote or estimate of maximum cotton yield, *b* is the estimate of the redroot pigweed density at which 50% yield loss occurs, and *D* is the weed density per meter of crop row.

Coefficients of determination (*r* ^*2*^) were calculated for nonlinear regressions and used to determine the goodness of fit to nonlinear models. All probabilities were two-tailed, and the significance level was set at *P* = 0.05. Analysis was performed with the statistical software SPSS 13.0.

## Results and Discussion

### Plant height and stem diameter

**Fig 1:**
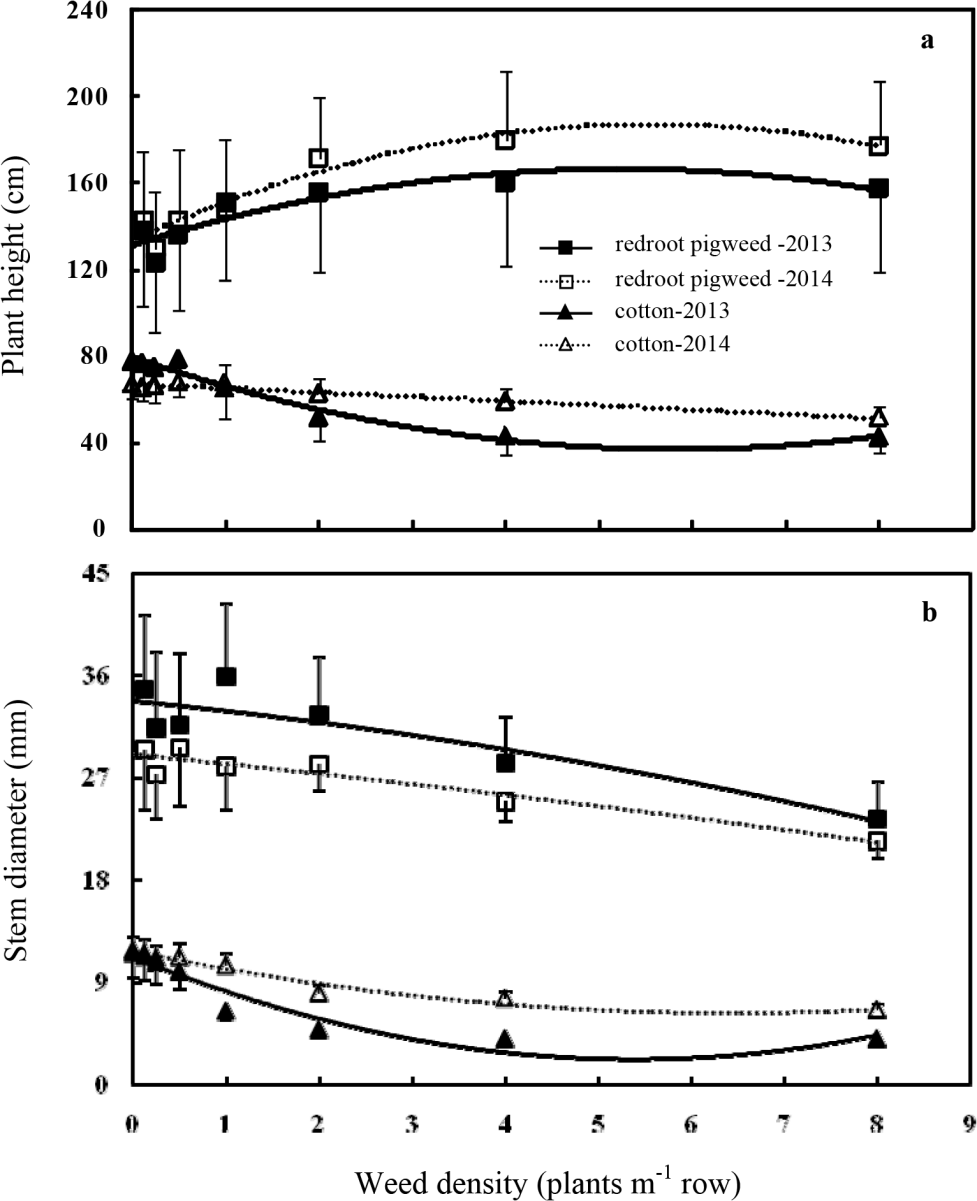
Relationship between redroot pigweed density and plant height (a) and stem diameter (b) of cotton and redroot pigweed. Plant height and stem diameter data were averaged over the growing season, and vertical bars indicate one standard error of the mean. Estimated parameters for these functions and for 2013 and 2014 are given in Table 2.

Plant height and stem diameter of redroot pigweed and cotton changed with the weed density and fit the quadratic functions well (Fig 1; Table 2). The mean height of the redroot pigweed plants increased at the high densities (1, 2, 4, and 8 plants m^-1^ of row). Conversely, the height of the cotton plants decreased through shading and competition from the weed especially under the competition of the weed above a density of 1 or 2 plants m^-1^ of row. Redroot pigweed at high densities resulted in strong shading of the cotton plants and prevented them from reaching their typical height; this is consistent with the competitive effect of velvetleaf (*Abutilon theophrasti*) on cotton observed by Cortés *et al*. [36]. The impact of increasing weed densities on the stem diameters followed similar trend in both cotton and redroot pigweed. Stem diameter of redroot pigweed and cotton decreased with increasing plant density. In summary, the intraspecific competition among weed plants resulted in the tall, slender redroot pigweed and the interspecific competition between cotton and weed created the thin, short cotton at the high weed densities. Average reductions of cotton plant height in 2013 and 2014 were approximately 13 and 2 cm, respectively, for each redroot pigweed plant m^-1^ of row according to regression equations, and redroot pigweed height increased about 12–18 cm for each redroot pigweed plant m^-1^ of row. Stem diameter reduction over the 2 yr averaged approximately 2 and 0.8 mm for each redroot pigweed plant m^-1^ of row for cotton and redroot pigweed, respectively.

**Table 2:** Parameter estimates for functions describing the effect of redroot pigweed density (*D*) or days after planting (*T*) on plant height or stem diameter (*Y*) of cotton and redroot pigweed.

Parameters	Species	Year	*Y* = *a* + *bD* + *cD* ^*2*^				$Y=a\;e^{be^{kT}}$			
			*a*	*b*	*c*	*r* ^*2*^	*a*	*b*	*k*	*r* ^*2*^
Plant height/cm	Cotton	2013	79.6	-14.6	1.256	0.963	106.5	-10.6	-0.039	0.995
		2014	67.2	-2.0	-0.005	0.947	77.6	-30.3	-0.071	0.997
	Redroot pigweed	2013	131.2	13.5	-1.288	0.786	257.3	-13.3	-0.047	0.995
		2014	133.3	19.4	-1.744	0.928	240.4	-17.7	-0.048	0.996
Stem diameter/mm	Cotton	2013	11.2	-3.3	0.309	0.923	12.5	-4.4	-0.029	0.990
		2014	11.7	-1.7	0.130	0.956	11.6	-11.9	-0.059	0.994
	Redroot pigweed	2013	33.8	-0.8	-0.065	0.816	42.4	-13.8	-0.063	0.992
		2014	29.1	-0.8	-0.018	0.905	33.8	-16.0	-0.061	0.985

**Fig 2:**
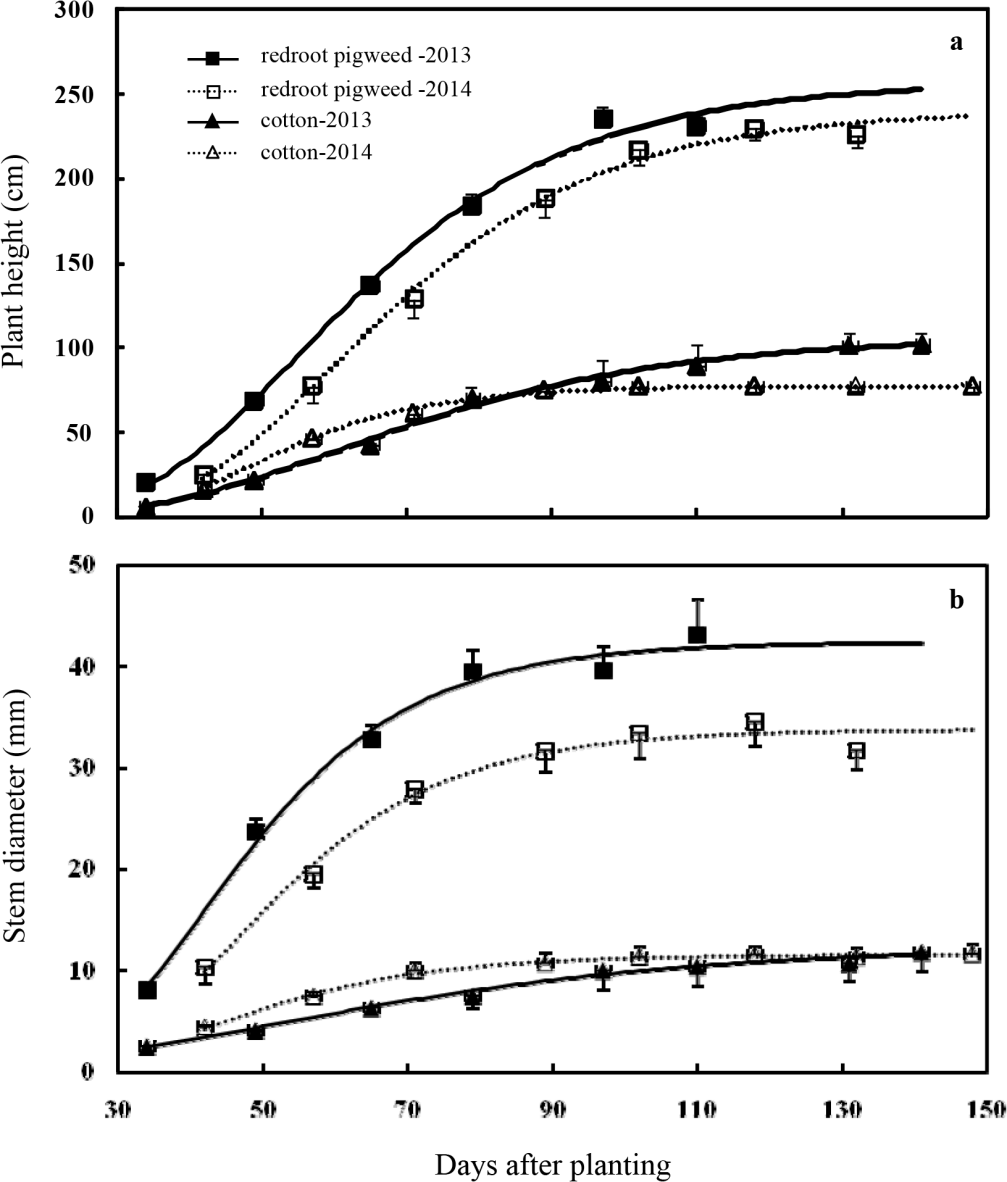
Predicted plant height (a) and stem diameter (b) of cotton and redroot pigweed over the growing season. Plant height and stem diameter data were averaged over weed densities, and vertical bars indicate one standard error of the mean. Estimated parameters for these functions and for 2013 and 2014 are given in Table 2.

Plant height and stem diameter of redroot pigweed and cotton over the season fit the Gompertz growth model well (Eq 1) when they were averaged over redroot pigweed densities (Fig 2; Table 2). Early vegetative growth is a key determination of crop: weed competition (e.g. [37]); consequently, redroot pigweed was taller and thicker than cotton and heavily shaded cotton throughout the season, which was the reason that redroot pigweed reduced cotton height at higher weed densities [38]. Other researchers had reported similar results that jimsonweed (*Datura stramonium*) growth was rapid early in the season and strong light interception occurred [39]. Because redroot pigweed growth was rapider than cotton during the whole growing season when weed plants emerged concurrent with cotton, it was necessary for effective weed management to apply soil-applied herbicides at planting. Although soil-applied herbicides seldom provided season-long control of weeds, they suppressed redroot pigweed plants for about one month and reduced the height advantage of weeds emerging late cotton.

### Cotton Reproductive Growth

**Fig 3:**
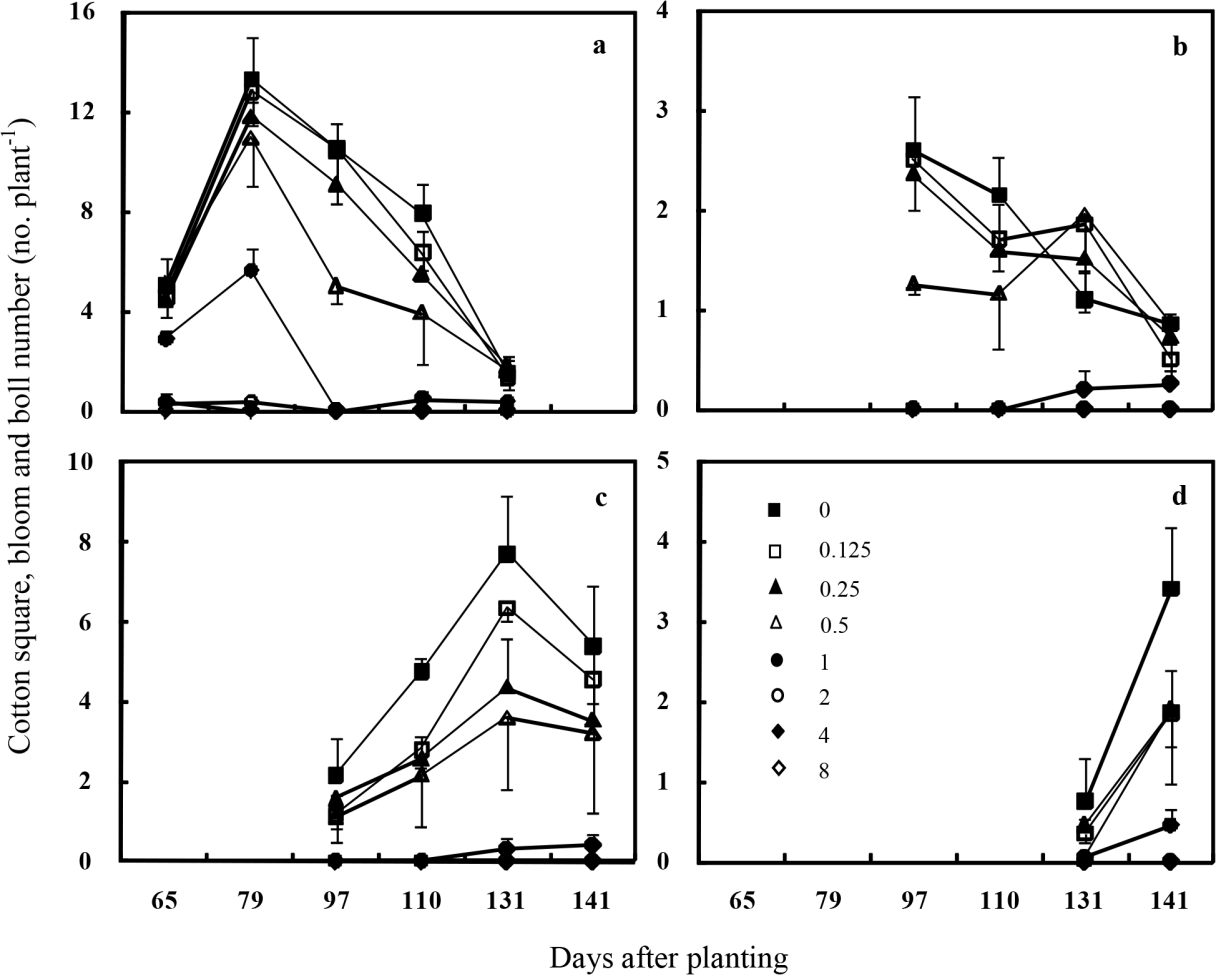
Seasonal variation of the number of cotton squares, blooms and bolls under different redroot pigweed densities in 2013 (a, square and bloom; b, small boll; c, large boll; d, cracked boll). **V**ertical bars indicate one standard error of the mean.

Reproductive growth of individual cotton plants was characterized by the number of squares, blooms, and bolls per plant, which were not combined because peak did not occur at the same time in each year and the total number at a given date varied between 2013 and 2014. In 2013, cotton squares and blooms were first observed in early July (65 DAP), with the exception of the maximum weed density treatment in which no squares or blooms were produced. However, there were different peaks under the competition of different weed densities. Peak cotton square and bloom counts occurred in mid July (79 DAP) when the redroot pigweed densities were less than 1 plant m^-1^ of row. When the weed density was at 2 plants m^-1^ of row, cotton squares and blooms could be observed only in July, and there was no longer any square or bloom in August (after 97 DAP). When the weed density increased to 4 plants m^-1^ of row, few cotton squares and blooms could be observed only in early July (Fig 3A). Cotton boll counts, including small bolls, large bolls, and cracked bolls, had different peaks due to the competition of redroot pigweed plants. Small bolls peaked at early August (97 DAP) in the weed-free, 0.125, and 0.25 plant m^-1^ of row treatments. Small boll peak was delayed to early September (131 DAP) in the 0.5 plant m^-1^ of row treatment. When the weed densities increased to 1 plant m^-1^ of row, small bolls did not appear until September (Fig 3B). Large bolls peaked at early September in the weed-free and 0.125–0.5 plant m^-1^ of row treatments, and the peak was delayed to mid September (141 DAP) when the weed density was at 1 plant m^-1^ of row (Fig 3C). The peak of cracked bolls was at mid September in the weed-free and 0.125–1 plant m^-1^ of row treatments (Fig 3D). Cotton boll was never observed during the growing season at the densities of 2, 4, and 8 weed plants m^-1^ of row, which was consistent with the 100% cotton yield loss in these treatments.

**Fig 4:**
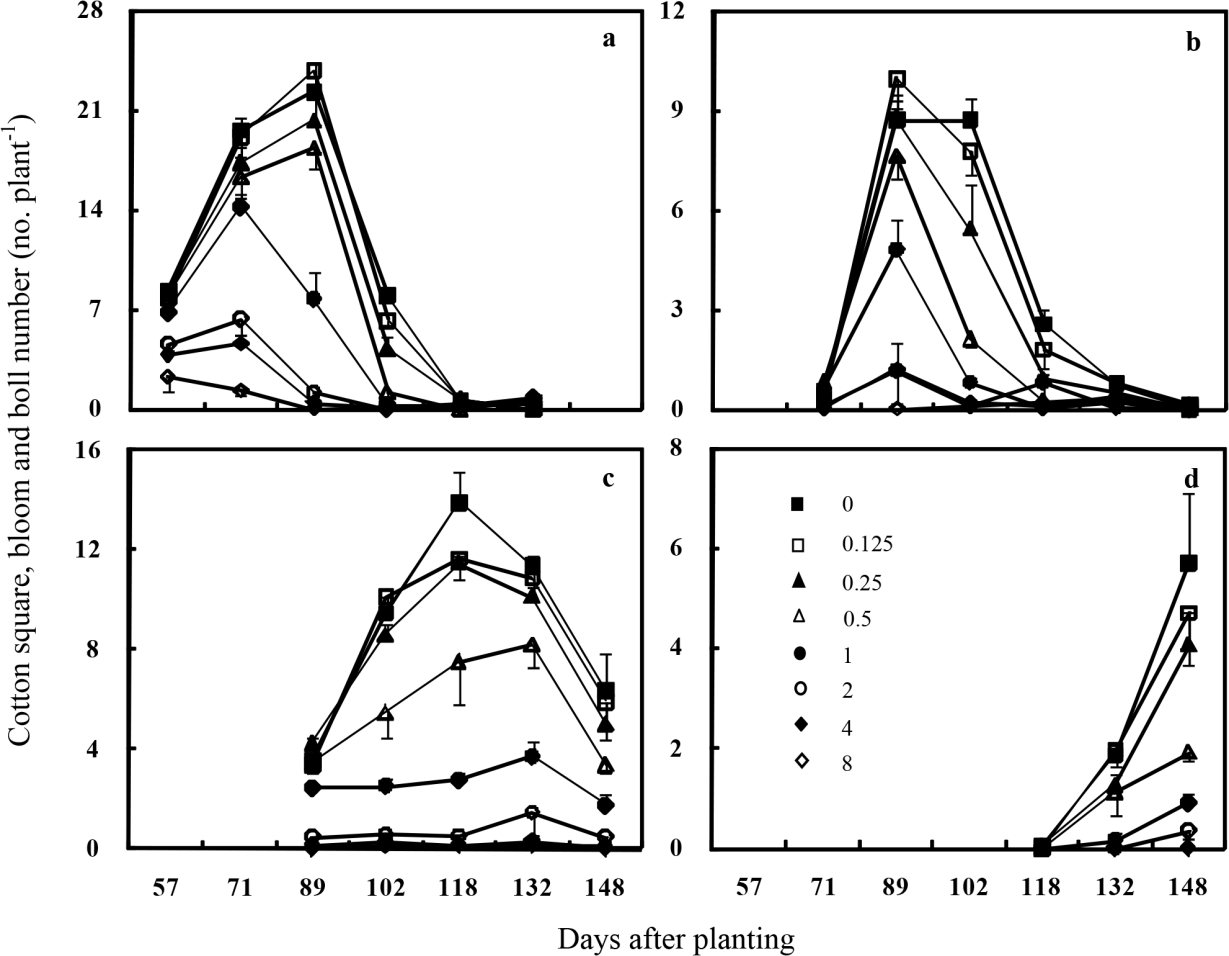
Seasonal variation of the number of cotton squares, blooms and bolls under different redroot pigweed densities in 2014 (a, square and bloom; b, small boll; c, large boll; d, cracked boll). **V**ertical bars indicate one standard error of the mean.

In 2014, there were still different peaks of cotton squares, blooms and bolls under the competition of different weed densities as the previous year. The emergence of cotton squares and blooms concentrated on July for all eight treatments. However, in the weed-free and 0.125–0.5 plant m^-1^ of row treatments, the peak of cotton squares and blooms occurred in late July (89 DAP). When the weed densities were at 1 to 8 plants m^-1^ of row, cotton squares and blooms peaked at mid July (71 DAP), and it declined rapidly at late July (Fig 4A). When weed densities were less than 2 plants m^-1^ of row, small bolls were firstly observed in mid July and peaked at late July. Small boll peak was not obvious in the 4 and 8 plant m^-1^ of row treatments (Fig 4B). Large bolls peaked from mid August to early September (102–132 DAP) in the weed-free and 0.125–0.5 plant m^-1^ of row treatments, but there were less large bolls without overt peak when the weed density was more than 4 plants m^-1^ of row (Fig 4C). As in 2013, cracked bolls mainly occurred during September (after 130 DAP), and the number of cracked bolls decreased with increasing of redroot pigweed density. Moreover, there was no cracked boll until late September (148 DAP) when weed density was at 4 and 8 plants m^-1^ of row (Fig 4D).

From the squares, blooms, and bolls data, it seemed that the interference of redroot pigweed could result in a delay in cotton maturity especially at the densities of 1 to 8 plants m^-1^ of row. Moreover, the weed density showed negative impact on square, bloom and boll numbers when the data were averaged across the season in each year. Results from this research were similar to previous studies. Barnett and Steckel [33] found that high densities of giant ragweed (0.8 and 1.6 plants m^-1^ of row) delayed cotton maturity which impacted final cotton lint yield. Because our primary objective was to determine the effects of redroot pigweed density on cotton growth under full season interference, we removed the weeds when they matured. In practice, redroot pigweed plants emerging before or concurrent with cotton should be controlled early in the growing season since reproductive growth of cotton had been affected as early as about 60 DAP when weed densities were at 1 to 8 plants m^-1^ of row (Figs 3 and 4). Moreover, with the extended germination period [15] of redroot pigweed, future studies should evaluate the effect of weed removal at various cotton growth stages. These studies will provide critical information for timely management of redroot pigweed.

### Seed Cotton Yield

**Fig 5:**
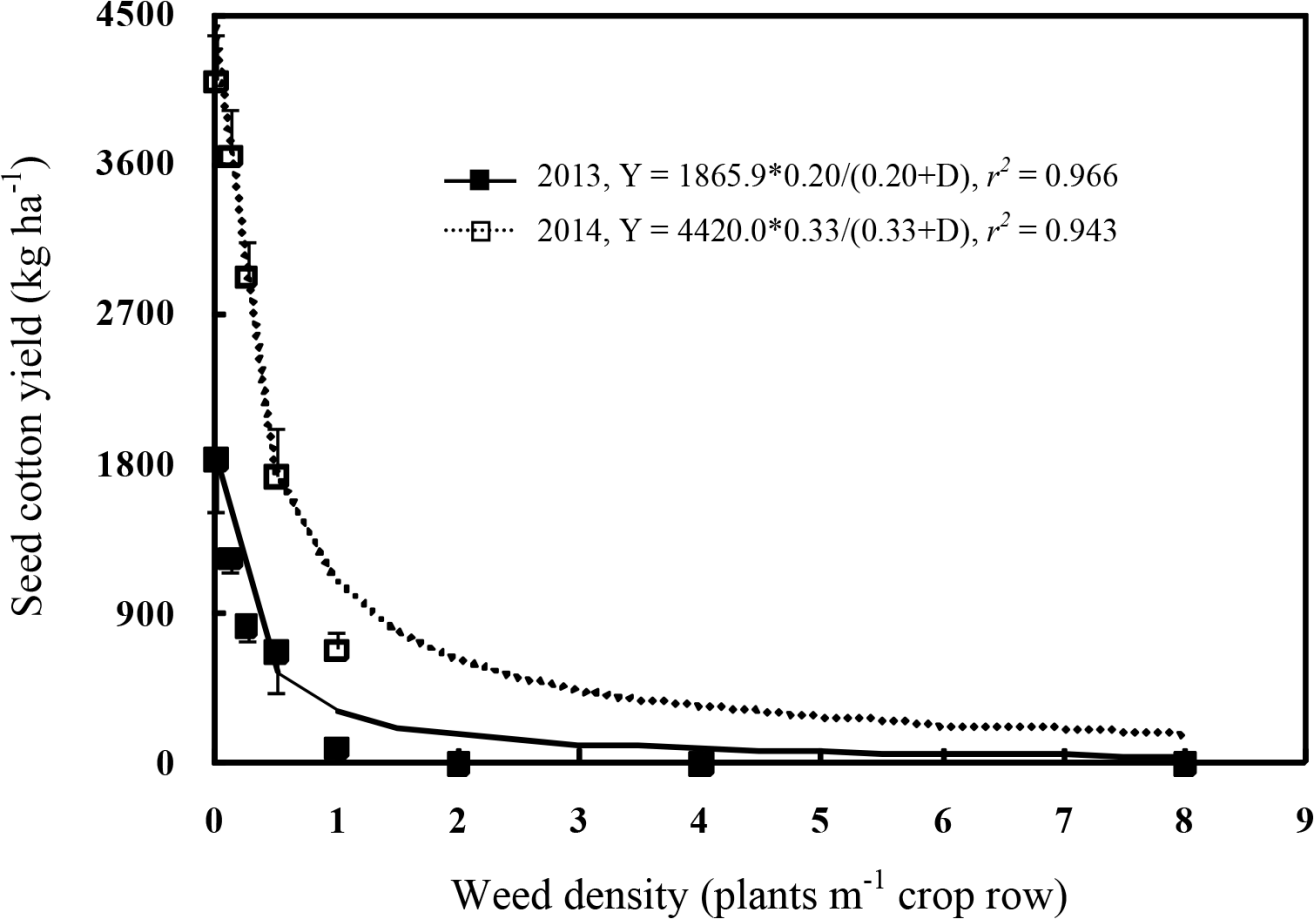
Cotton yield loss associated with increasing redroot pigweed density. Regressions are based on treatment means, and vertical bars indicate one standard error of the mean.

Seed cotton yield was closely associated to the density of redroot pigweed plants (Fig 5). Seed cotton yields with the weed-free treatment were 1,830 and 4,098 kg ha^-1^ in 2013 and 2014, respectively. As redroot pigweed density increased, seed cotton yield was reduced. The threshold density at which statistically significant yield reduction appeared was at 0.125 and 0.25 weed plant m^-1^ of row in 2013 and 2014, respectively, which reduced cotton seed yield by 28%. Higher densities of redroot pigweed resulted in greater yield reductions, and at 1 plant m^-1^ of row, yields were reduced 83–95%, more than 30% and 66% reduction by tumble pigweed [20] and Palmer amaranth [40], respectively. Two to eight redroot pigweed plants m^-1^ of row prevented production of any harvestable cotton.

The hyperbolic decay regression model (Eq 2) estimated that a density of 0.20–0.33 redroot pigweed m^-1^ of row would result in a 50% seed cotton yield loss from the maximum yield in this study. Previous studies reported that Palmer amaranth needed 0.38–0.87 plant m^-1^ of row to reduce cotton yield by 50% [22, 40]. Barnett and Steckel [33] reported that a density of 0.26 giant ragweed plant m^-1^ of row would result in a 50% cotton yield loss. Snipes *et al*. [30] found that 0.37–0.53 common cocklebur (*Xanthium pensylvanicum*) plant m^-1^ of row was needed to reduce cotton yield by 50%.

### Cotton yield components

Cotton bolls were not developed during the growing season at the weed densities of 2, 4, and 8 weed plants m^-1^ of row. Data of boll weight, seed number, lint percentage and seed index were therefore unavailable for these three treatments. Redroot pigweed at various densities did not have significant effects on cotton lint percentage and seed index, while the boll weight and seed numbers per boll were significantly affected. In both years, redroot pigweed at densities of 0.5 weed m^-1^ of row or greater caused significant reductions in boll weight (13.3–30.2%) and seed numbers per boll (7.8–20.4%) when compared with the weed-free control (Table 3).

**Table 3:** Influence of redroot pigweed densities on cotton yield components.

Redroot pigweed density	Boll weight		Seed numbers		Lint percentage		Seed index	
	2013	2014	2013	2014	2013	2014	2013	2014
# m^-1^ row (# m^-2^)	g		# boll^-1^		%		g	
0	6.3±0.03 a*	7.5±0.15 a	35.8±0.14 a	37.3±0.61 ab	37.4±0.15 a	43.1±0.45 a	10.8±0.03 a	11.4±0.21 a
0.125 (0.16)	5.7±0.00 ab	7.2±0.23 a	35.3±0.26 ab	38.0±1.24 a	35.3±0.41 a	44.8±0.85 a	10.4±0.02 a	10.6±0.20 a
0.25 (0.31)	5.6±0.13 ab	7.1±0.09 a	33.7±0.48 abc	37.1±0.86 ab	37.6±0.42 a	44.8±0.52 a	10.8±0.12 a	10.7±0.29 a
0.5 (0.63)	5.2±0.62 bc	6.5±0.12 b	30.0±2.51 c	34.4±0.77 b	36.8±0.82 a	42.7±0.79 a	10.6±0.57 a	10.9±0.20 a
1 (1.25)	4.4±0.44 c	5.6±0.32 c	31.6±1.18 bc	29.7±1.25 c	36.9±0.51 a	42.2±1.90 a	8.9±0.83 a	10.4±0.30 a

* Means ± SE within columns followed by the same letter are not significantly different between treatments at the 0.05 probability level as determined by Fisher’s Protected LSD test.

Seed cotton yield tended to decrease as the redroot pigweed density increased in both experimental years. It may be a result of the cotton plants being under severe weed competition and producing fewer and smaller boll than normal, and thus decreasing seed cotton yield. Other researchers had reported similar results that yield reduction caused by weed competition was primarily a result of reduction in cotton boll number and weight [41–43]. So, boll numbers per cotton plant and boll weight are very important determinants of cotton yield [43]. Both boll weight and seed number per boll were reduced to some extent in some of the redroot pigweed competition treatments while the lint percentage was not affected by redroot pigweed competition, which was consistent with the earlier studies [9, 34, 44]. The cotton seed index was also not affected by redroot pigweed competition, which was in agreement with Buchanan and Burns [34]. In conclusion, the redroot pigweed interference could reduce cotton boll weight and seed number per boll, but did not affect the lint percentage or seed weight.

### Cotton fiber quality

Cotton fiber quality was evaluated to determine if redroot pigweed densities affected fiber length, fiber length uniformity, micronaire, breaking elongation and fiber strength of the hand-harvested samples. Results from our studies indicated that redroot pigweed did not affect any evaluated fiber quality characteristic, except for the fiber length uniformity and micronaire in 2014, which were significantly reduced at the density of 1 redroot pigweed plant m^-1^ of row (Table 4). This result was similar to earlier reports that fiber quality traits are not as sensitive as cotton yield in assessing weed interference effects [20, 21, 33, 44, 45]. However, other studies have indicated that certain weed species, including ivyleaf morningglory (*Ipomoea hederacea*), hogpotato (*Hoffmanseggia glauca*), unicorn-plant (*Proboscidea louisianica*), and johnsongrass (*Sorghum halepense*), could reduce fiber quality at high densities [32, 42, 46, 47].

**Table 4:** Influence of redroot pigweed densities on cotton fiber quality.

Redroot pigweed density	Fiber length		Length uniformity		Micronaire		Breaking elongation		Fiber strength	
	2013	2014	2013	2014	2013	2014	2013	2014	2013	2014
# m^-1^ row (# m^-2^)	mm		%		unit		%		cN tex^-1^	
0	27.9±0.13 b*	30.4±0.30 a	83.1±0.54 a	84.8±0.37 a	4.5±0.24 a	5.6±0.10 a	6.6±0.06 a	6.2±0.02 a	27.3±0.38 a	29.8±0.41 a
0.125 (0.16)	27.7±0.20 b	29.8±0.55 a	82.9±0.40 a	83.3±0.50 b	4.7±0.02 a	5.7±0.04 a	6.6±0.03 a	6.2±0.02 a	27.1±0.43 a	28.6±0.34 a
0.25 (0.31)	28.7±0.25 a	29.6±0.19 a	84.1±0.46 a	84.8±0.09 a	4.6±0.08 a	5.3±0.23 a	6.5±0.06 a	6.3±0.02 a	27.1±0.16 a	29.4±0.50 a
0.5 (0.63)	27.6±0.11 b	29.9±0.25 a	83.4±0.26 a	85.2±0.71 a	4.7±0.07 a	5.2±0.15 ab	6.7±0.09 a	6.2±0.02 a	27.1±0.26 a	29.4±0.22 a
1 (1.25)	27.8±0.09 b	29.9±0.33 a	82.8±0.43 a	83.1±0.11 b	4.5±0.09 a	4.7±0.28 b	6.7±0.06 a	6.3±0.00 a	27.0±0.30 a	28.5±0.48 a

* Means ± SE within columns followed by the same letter are not significantly different between treatments at the 0.05 probability level as determined by Fisher’s Protected LSD test.

### Weed biomass

**Fig 6:**
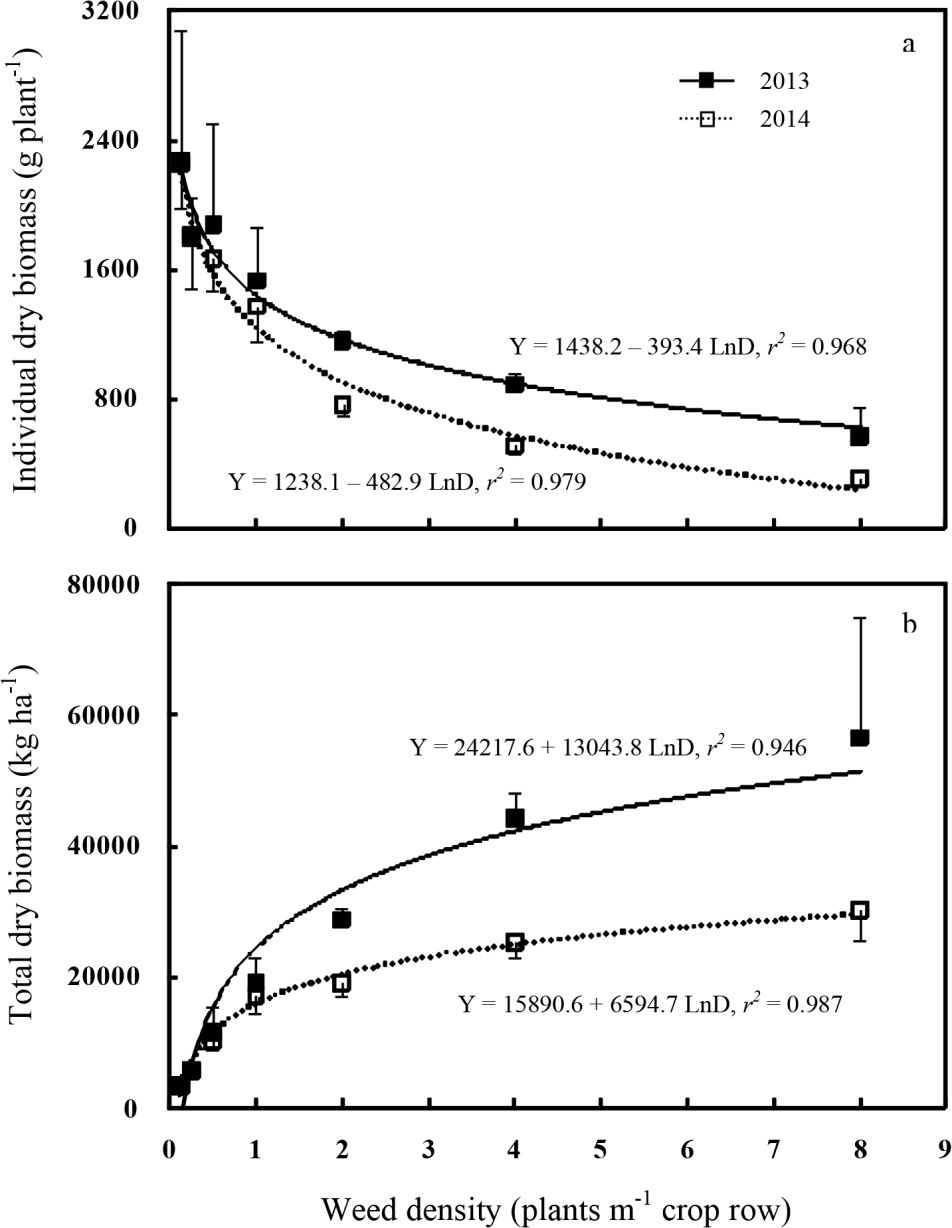
Relationship between redroot pigweed density and its dry biomass. Regressions are based on treatment means, and vertical bars indicate one standard error of the mean.

Redroot pigweed dry biomass at the end of the growing season was also evaluated. Individual redroot pigweed dry weights decreased as plant density increased, and the relationship between individual redroot pigweed dry biomass and weed density was logarithmic, and year effects were significant (Fig 6A). Weed biomass combined over the two experiments showed a reduction from 2,257 g plant^-1^ at the density of 0.125 plant m^-1^ of row to 434 g at 8 plants m^-1^ of row. The density-dependent effects on weed biomass per plant indicated that intraspecific competition occurred in the range of densities evaluated. In other studies with similar weed density ranges, increasing plant density also reduced dry biomass of weeds in cotton, including common cocklebur [30], redroot pigweed [38], jimsonweed [39], and buffalobur (*Solanum rostratum*) [45].

Regression analysis showed that redroot pigweed biomass ha^-1^ tended to increase with increasing weed density and the results also fit logarithmic model well (Fig 6B). The lowest density of 0.125 redroot pigweed plant m^-1^ of row produced about 3,500 kg ha^-1^ of dry matter in both years. The highest density of 8 redroot pigweed plants m^-1^ of row produced dry biomass ranging from 30,200 to 56,500 kg ha^-1^. Increasing weed density from 0.125 up to 8 plants m^-1^ of row resulted in biomass production of redroot pigweed that was only 9–16 times higher than in the treatment with 0.125 weed plant m^-1^ of row. It could be explained by the fact that intraspecific competition between redroot pigweed plants at low weed density was not so stronger than at higher density.

### Weed seed production

**Fig 7:**
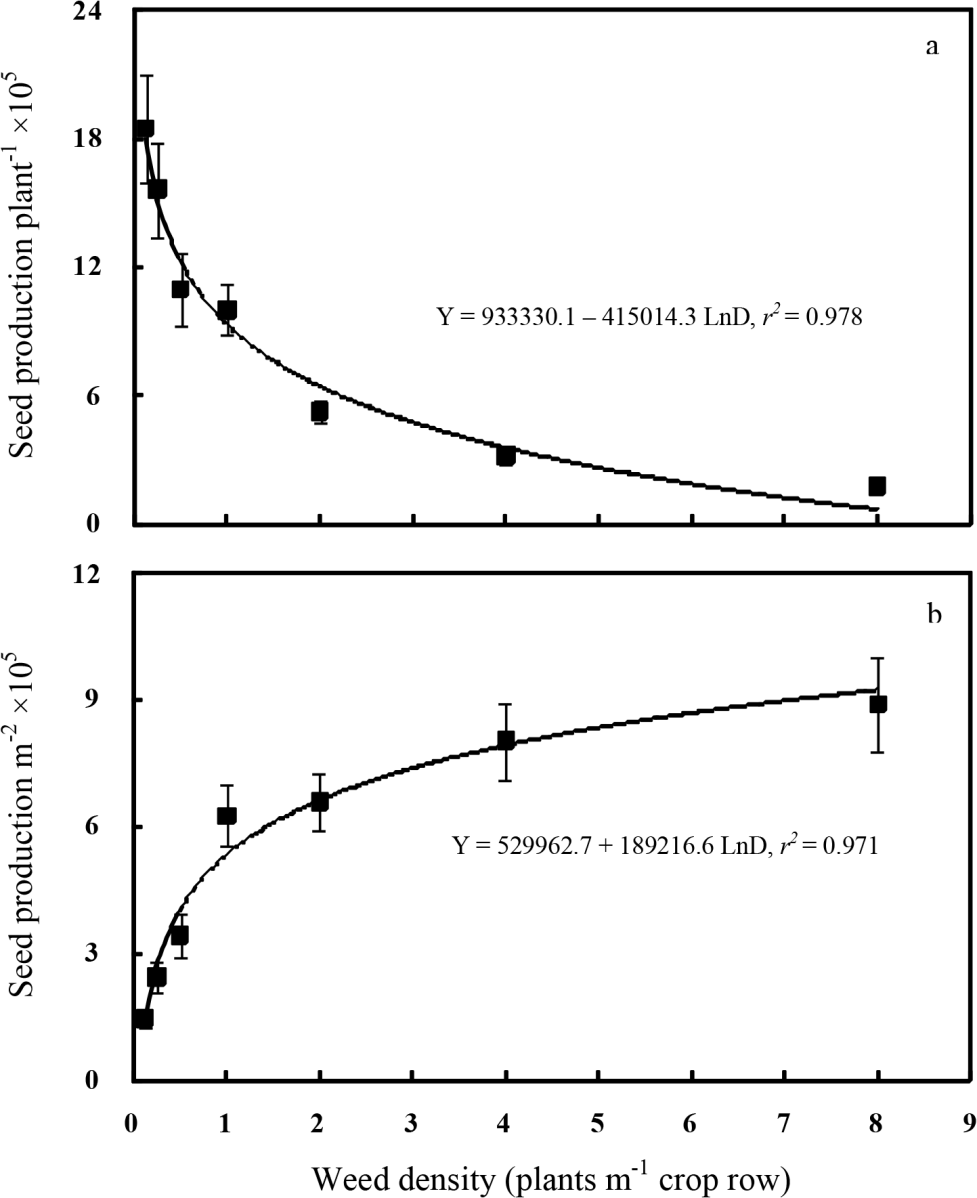
Redroot pigweed seed production per plant (a) or per square meter (b) as a function of plant density in 2014. Regressions are based on treatment means, and vertical bars indicate one standard error of the mean.

Weed seed production per plant was density dependent as indicated by a logarithmic response (Fig 7A). Individual redroot pigweed seed production decreased as weed density increased and reduced from 1,846,000 at 0.125 plant m^-1^ of cotton row to 178,000 at 8 plants m^-1^ of row. This result could be attributed to the more intraspecific competition between redroot pigweed plants at higher weed density. The previous studies also reported that seed production per plant for redroot pigweed decreased as plant density increased [2, 3]. Seed production of redroot pigweed is enormous; Mohler and Callaway [48] reported that plants of redroot pigweed produce up to 100,000 seeds per plant in the absence of any crop or herbicide. Knezevic and Horak [12] reported that redroot pigweed was capable of producing prodigious amount of seed up to 400,000 seeds per plant.

The number of redroot pigweed seed production per square meter increased with increasing weed density and also fit a logarithmic function well (Fig 7B). At a density of 1 plant m^-1^ of cotton row, redroot pigweed produced 626,000 seeds m^-2^. By comparison, ladysthumb (*Polygonum persicaria*) [26], Pennsylvania smartweed (*P*. *pensylvanicum*) [27], pale smartweed (*P*. *lapathifolium*) [28], unicorn-plant [32], and jimsonweed [39], at 1 plant m^-1^ of cotton row produced 40,000, 22,000, 44,000, 4,700, and 23,000 seeds m^-2^, respectively. Bensch *et al*. [5] observed that seed number m^-2^ of redroot pigweed in competition with soybean was < 20,000 seeds m^-2^ at different densities. Amini *et al*. [3] found that redroot pigweed could produce 16,000–52,000 seeds m^-2^ in competition with different red kidneybean (*P*. *vulgaris*) cultivars. In this study the range of seed number m^-2^ for redroot pigweed in competition with cotton was obtained 144,000–890,000 seeds m^-2^ at different weed densities. The higher quantity of seeds in our study may be due to longer growing season at our study site or fertility differences in crop production of soybean, red kidneybean vs. cotton.

Weed seed production is a concern of growers and other agricultural personnel in order to improve weed management or crop production [26–28]. Results from this study demonstrated that seed production of redroot pigweed is prodigious. High fecundity and prolonged seed longevity [13, 14] make seed bank management difficult and make the eradication of redroot pigweed nearly impossible. Therefore, growers need to control redroot pigweed early in the growing season to avoid seed producing and reduce seed bank of weed.

## Conclusions

Present study demonstrated that redroot pigweed is a highly competitive weed which affects cotton growth and development and decreases cotton yield even at low densities. It is known that competitiveness of weeds with cotton depends on weed morphology [9, 34], its phenology [49] and its differential response to environmental factors such as light, water and nutrients [50–52]. Redroot pigweed is a vigorous plant that can reach a height of 3 m and then it is always able to gain a height advantage over the cotton and have the advantage of spreading over the top of the cotton canopy. The height advantage during the competition between crop and weed has also been shown by other studies [3, 53]. Meyers *et al*. [54] reported that the seed yield reduction of sweetpotato (*I*. *batatas*) in competition with Palmer amaranth was attributed to increased height and subsequently more light interception by the weed. Additionally, weed canopy above crop could affect the light quality (i.e. red to far-red light ratio) intercepted by crop plants, thereby affecting their growth and competitiveness [55, 56]. In contrast, inability to shade cotton and compete for light early in the growing season contributed to poor competitiveness of bermudagrass (*Cynodon dactylon*) [57], ladysthumb [26], Pennsylvania smartweed [27], and pale smartweed [28] with cotton. Therefore, short weeds appear to be less damaging to cotton than the tall weeds [31]. Furthermore, the small amount of light reaching infested cotton plants reduced their water and nutrient use capacity [58, 59], and then redroot pigweed plants used more of these resources, making less available for cotton. Competition for water and nutrients is frequently credited with reduced crop yield [20, 29, 42].

Although the effects of redroot pigweed density on phenotypic traits of weed and cotton, including vegetative and reproductive growth indices and quantity and quality characteristics, were described clearly in this study, physiological mechanisms of the competition between weed and crop should consider more than just morphological features. Future work should address effects on leaf area index, photosynthetically active radiation, and growth rate, which are the better growth indices involved in competitiveness of crop against weed [3, 6].

## Supporting Information

S1 Dataset Raw data for Tables 1, 3, and 4 and Figs 1–7. (XLS)
